# Editorial: New insights into the mechanisms of resistance to anti-cancer drugs

**DOI:** 10.3389/fonc.2022.990144

**Published:** 2022-07-26

**Authors:** Francesco Bertoni, Simona Rapposelli

**Affiliations:** ^1^ Institute of Oncology Research, Faculty of Biomedical Sciences, Università della Svizzera Italiana (USI), Bellinzona, Switzerland; ^2^ Oncology Institute of Southern Switzerland, Ente Ospedaliero Cantonale (EOC), Bellinzona, Switzerland; ^3^ Department of Pharmacy, University of Pisa, Pisa, Italy

**Keywords:** chemoresistance, anti-cancer drugs, targeted-therapy, drug resistance, combination therapy

The development of resistance after an initial response by tumor cells to anti-cancer therapies is perhaps one of the main causes that lead cancer patients to succumb to their disease. Resistance can develop against any type of therapies, including chemotherapy, targeted agents (small molecules or antibody-based) and cellular therapies. Several biological mechanisms are involved in drug resistance and include increased drug efflux, changes in drug metabolism, alterations in drug target interaction, evasion of apoptosis, and activation of alternative signaling pathways. Unfortunately, this high heterogeneity in response to treatments and the complexity of drug resistance, including the fact that multiple resistance mechanisms might be active at the same time in an individual patient, make it difficult to achieve complete responses and avoid tumor progressions and relapses. This Research Topic collects the latest data on mechanisms responsible for the onset of chemoresistance and presents new combination therapies to overcome the mechanisms of resistance in different types of cancer.

Two contributions are focused on triple-negative breast cancer, one of the tumors still much in need of effective therapies. Wu et al. explore modalities directly targeting the tumor cells, combining the mTOR inhibitor rapamycin with itraconazole, a broad-spectrum antifungal agent with also anti-tumor activity, achieving synergism although only in terms of increased cell cycle arrest but not in increased induction of cell death. Ghallab et al. instead explore a therapeutic modality targeting the interaction between the tumor cell and the tumor microenvironment. After studying CXCR2 and TFGbeta expression pattern and potential role in sustaining resistance to the chemotherapy agent doxorubicin, Ghallab et al. use AZD5069, a CXCR2 antagonist small molecule, to counteract this feedback and also to improve, at least in an *in vitro* system, the response to the anti-PDL-1 immune checkpoint modulator atezolizumab.

Three contributions are devoted to lung cancer. Zeng et al. describe two cases treated with the EGFR inhibitor osimertinib focusing on the genetic events preceding, and, especially, following the treatment with the tyrosine kinase inhibitor that might be involved in resistance to this targeted agent. The paper by Zeng et al. is on the role of glycosylation, and in particular of the resistance to cisplatin, and how, in cellular models of non-small cell lung cancer, the glycosylation inhibitor tunicamycin can reduce the chemoresistance. Xiang Li et al. look at the copper chelator ammonium tetrathiomolybdate (ATTM), used for the treatment of hereditary copper metabolism conditions and with possible anti-cancer properties.

In lung adenocarcinoma cells, the Authors observed that hydrogen sulfide, induced by the exposure to ATTM, might impede the anti-tumor activity of ATTM itself. Interestingly, hydrogen sulfide is also the topic of another work of this Research Topic. Indeed, Mao et al. provide evidence on the negative impact of hydrogen sulfide on the anti-tumor activity of the thioredoxin inhibitor PX-12.

Hu et al. have contributed to this issue with a comprehensive overview on the role of non-coding RNAs in sustaining the resistance to the multi-kinase inhibitor sorafenib in hepatocellular carcinoma, showing how non-genetic also contribute to the reduced activity of anti-cancer agents.

Finally, two contributions are on hematological cancers. Goel et al. use an *in vivo* model of T-cell lymphoma, namely the murine thymus-derived Dalton’s Lymphoma to characterize the anti-tumor activity of the natural oxylipin methyl jasmonate, which appears to modulate the expression of various genes involved in drug resistance. Finally, Zhang et al. applied computer modeling to tackle the changes induced by the asciminib (ABL001), an allosteric BCR-ABL1 inhibitor, on BCR-ABL1 itself in its mutant and wild-type forms, to optimize co-administration of orthosteric tyrosine kinase inhibitors and allosteric drugs for chronic myeloid leukemia patients.

## Author contributions

All authors listed have made a substantial, direct, and intellectual contribution to the work and approved it for publication

## Conflict of interest

The authors declare that the research was conducted in the absence of any commercial or financial relationships that could be construed as a potential conflict of interest.

## Publisher’s note

All claims expressed in this article are solely those of the authors and do not necessarily represent those of their affiliated organizations, or those of the publisher, the editors and the reviewers. Any product that may be evaluated in this article, or claim that may be made by its manufacturer, is not guaranteed or endorsed by the publisher.

